# It Will Out

**Published:** 1882-05

**Authors:** 


### IT WILL "OUT."

IN the reign of Louis XIV. a certain brilliant abbé was one of a large party who had assembled round the royal supper table. There were clever talkers, sharp dealers in epigram, skillful bandiers of compliments and repartee. One lady, famous for her wit, being asked to name the three sights that gave her the greatest pleasure, replied: "A great general on a war horse, a great preacher on a platform and a great thief on a gallows." The abbé added to the mirth of the evening by telling of the adventures of a gay and memorable career. "I remember," he said, "very well the first penitent who ever came to my confessional. I was young then and little accustomed to hear the secrets of court life. It was a murderer who told me the secret of his crime." The abbé was pressed to tell the tale or to give a clue to the culprit; but he kept a guarded and wary silence. Presently in came one of the most trusty of the king's favorites. "Ah! M. l'Abbé," he said, recognizing an old friend, "gentlemen, I was the first penitent whom the abbé ever shrived, and I promise you when I told him my story, he heard what astonished him!" That night the nobleman was carried to the Bastile, and the evidence of a crime committed thirty years before was complete and the culprit detected.

### A TEMPERANCE SERMON.

A stronger temperance sermon will never be preached than that which an unfortunate woman of Cape Girardeau, Mo., recently delivered before her husband in a bar-room. Setting a covered dish, which she had brought with her, upon a table, she said: "Presuming, husband, that you are too busy to come home to dinner, I have brought you yours," and departed. With a forced laugh he invited his friend to dine with him; but, on removing the cover from the dish, found only a slip of paper, on which was written: "I hope you will enjoy your meal; it is the same as your family have at home."

